# Preparation of a Pd/Al_2_O_3_ Catalyst with Microwave-Induced Plasma Jet Irradiation under Atmospheric Pressure

**DOI:** 10.3390/nano9121734

**Published:** 2019-12-05

**Authors:** Jai Young Chung, Satoshi Kodama, Hidetoshi Sekiguchi

**Affiliations:** Department of Chemical Science and Engineering, School of Materials and Chemical Technology, Tokyo Institute of Technology, Meguro-ku, Tokyo 152-8550, Japan; skodama@chemeng.titech.ac.jp (S.K.); hsekiguc@chemeng.titech.ac.jp (H.S.)

**Keywords:** microwave-induced plasma, Pd/Al_2_O_3_ catalyst, acetylene hydrogenation

## Abstract

Microwave-induced plasma under atmospheric pressure is an effective technique for catalyst preparation. A Pd/Al_2_O_3_ catalyst was prepared using a fixed bed with microwave plasma irradiation. The activity of the catalyst was compared with that of catalysts made using the plasma spouted bed and the conventional furnace. From the results of X-ray powder diffraction (XRD) spectra and transmission electron microscopy (TEM) images, plasma treatment induced a rapid reduction process (PdO→Pd). Moreover, the plasma treatment derived the growth of a different facet from Pd (111) to Pd (100). A different kind of phase transition behavior was observed with plasma-treated alumina. H_2_ chemisorption analysis confirmed that the plasma treatment had a positive effect on the dispersion of Pd metal on the support. These improvements to the properties of the catalyst resulted in excellent performance in hydrogenation of acetylene.

## 1. Introduction

Plasma, considered as the fourth state of matter, is a partially ionized gas that contains excited species as radicals. Plasma has been introduced into various catalyst preparation processes as an alternative to conventional thermal treatment [1,2,3,4,5,6,7]. Various types of plasma, including dielectric barrier discharge, radio frequency, and microwave-induced plasma, have been applied in catalyst modification, and remarkable advantages have been observed. For example, performance has improved, which has saved preparation time and enhanced energy efficiency.

Dielectric barrier discharge (DBD) plasma of Ar/H_2_ has been applied to reduce the precursors of the Ni/γ-Al_2_O_3_ and CeO_2_-Ni/γ-Al_2_O_3_ catalysts, which show high reactivity in dry reforming and durability in coke resistance [7]. Plasma treatment results in production of catalysts that possess different crystal structures, which could yield an improvement in carbon resistance [6]. Generally, different crystal structures in a metal show different behaviors in catalytic performance for specific catalytic reactions [8,9,10,11,12]. Microwave plasma has been applied for Ag/Al_2_O_3_ catalyst preparation under vacuum conditions. With this intervention, higher nitric oxide (NOx) reduction efficiency in the selective catalytic reduction with ethanol was observed when compared to that of calcined catalysts [5]. Plasma treatment for catalysts could enhance metal and support interactions [6,7]. In particular, a microwave-induced plasma jet combined with a spouted bed demonstrated excellent performance of Pd/Al_2_O_3_ in the selective hydrogenation of acetylene to ethylene in our previous research [13]. Microwave-induced plasma could create an environment of high temperature as a non-equilibrium plasma, and it could provide active species such as radicals and substances under atmospheric pressure. The spouted bed reactor could provide high mass and heat transfer between gases and solids [14,15]. A combination of plasma and the spouted bed could provide effective circumstances for various particle treatments. The results showed that the plasma spouted bed had beneficial effects on the size of Pd nanoparticles and on the interaction between Pd metal and an alumina support. In addition, a synergetic effect of the plasma and the spouted bed was observed. However, it remains unclear exactly how the plasma spouted bed could affect the properties of catalysts due to its multiple effects on catalyst preparation. Therefore, we focused on the effects of microwave-induced plasma irradiation alone on the catalyst. A Pd/Al_2_O_3_ catalyst was prepared using a fixed bed with microwave-induced plasma jet irradiation, and the activity of the catalyst was compared with that of catalysts made using the plasma spouted bed and a conventional furnace.

## 2. Experimental

### 2.1. Experimental Setup

**Figure 1:**
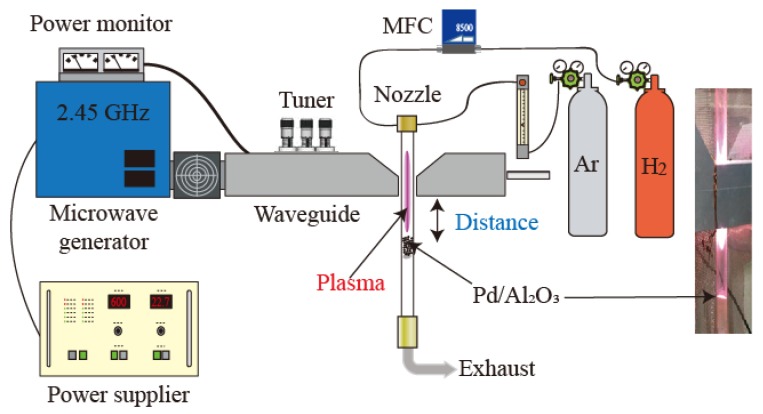
Experimental setup of the fixed bed with the plasma irradiation. A schematic diagram of the reactor and a photograph of the real apparatus (right).

Figure 1 shows a diagram of the fixed-bed reactor with the microwave-induced plasma jet. Microwave power generated by a 2.45 GHz microwave generator (IDX Corp. ING-25, Tokyo, Japan) was supplied to a quartz glass tube through a waveguide. The fixed bed consisted of a quartz glass tube with an internal diameter of 9 mm and an outer diameter of 11 mm. A mixture of Ar and H_2_ was used as a plasma gas for the reduction of the Pd precursor. Each gas nozzle was connected tangentially to the quartz tube. Since the tangential gas flow induced an annular swirl gas flow, the minimum pressure could be localized to the center of the tube due to centrifugal forces, resulting in stable plasma [16]. The catalyst precursor was set in the middle of the quartz tube below the plasma generator. For the conventional thermal treatment, a ceramic tube furnace (ARFLC-30KC, Asahi Rika Seisakusho, Chiba, Japan) with a maximum power of 600 W was used for catalyst preparation. The experimental setup and conditions of the plasma spouted bed were illustrated in our previous research [13].

### 2.2. Sample Preparation

As mentioned above, three preparation methods were applied for the Pd/Al_2_O_3_ catalyst: a fixed particle bed with plasma irradiation (PF), a plasma spouted bed reactor (PS), and the conventional method (CM) [17,18,19,20]. The precursor of the Pd/Al_2_O_3_ catalyst containing 1 wt% of Pd was prepared according to the following procedure:

<Step 1> Al(OH)_3_ powder (gibbsite) was added into 5 wt% of Pd(NO_3_)_2_ solution.
<Step 2> The solution was dried at 80 °C until it became slurry-like.
<Step 3> The slurry was dried at 110 °C for 12 h and crushed.

The crushed particles were 250–595 μm in size. The crushed particles were then treated by three preparation methods, namely the PF, the PS, and the CM, as follows:

<Step 4 (PF)> The crushed particles were treated in the plasma using the fixed bed under the following plasma conditions: power = 270 W, Ar flow rate = 2.5–4 L/min, H_2_ flow rate = 60 mL/min, and treatment time = 15 min.
<Step 4 (PS)> The crushed particles were treated using the plasma spouted bed reactor with the same plasma conditions as those used in PF.
<Step 4 (CM)> In the same way as in PF, the crushed particles were heated to 500 °C or 900 °C using the electric furnace with a 16.7% H_2_/Ar mixture for 2 h.

Details of the treatment conditions for the PF, the PS, and the CM are shown in Table 1. Table 1 illustrates that the distance between the microwave waveguide and the particle bed was changed for PF, while the treatment temperature was changed for the CM.

### 2.3. Evaluation of the Pd/Al_2_O_3_ Catalyst: Selective Hydrogenation of Acetylene to Ethylene or Ethane

A stainless reactor of 6 mm id (internal diameter) with a temperature-programmed electric furnace was used for the acetylene hydrogenation reaction. For each test, the reaction temperature was set to 120 °C, and 20 mg of the Pd/Al_2_O_3_ catalyst was added to the middle of the reactor. The gas was then fed into the reactor at flow rates of 20 mL/min (C_2_H_2_), 80 mL/min (H_2_), and 200 mL/min (Ar). The gas space velocity was 320,000 h^−1^. The gas that was produced was extracted from the sampling port and quantitatively analyzed using a gas chromatograph (Shimadzu GC-8APF, Kyoto, Japan). Hydrogenation of C_2_H_2_ is a sequential reaction to produce C_2_H_4_ and C_2_H_6_. The experimental results indicated that the produced gas consisted of C_2_H_2_, C_2_H_4_, C_2_H_6_, and H_2_. Thus, to evaluate catalyst activity, C_2_H_2_ conversion and C_2_H_4_ and C_2_H_6_ selectivity were calculated as follows:

(1) $$C_{2}H_{2}\;\mathrm{Conversion}\;\left(\%\right)=\left(\frac{C_{2}H_{2}\left(\mathrm{Feed}\right)-C_{2}H_{2}}{C_{2}H_{2}\left(\mathrm{Feed}\right)}\right)\times 100$$

(2) $$C_{2}H_{4}\;\mathrm{selectivity}\;\left(\%\right)=\left(\frac{C_{2}H_{4}}{C_{2}H_{2}\left(\mathrm{Feed}\right){-C}_{2}H_{2}}\right)\times 100$$

(3) $$C_{2}H_{6}\;\mathrm{selectivity}\;\left(\%\right)=\left(\frac{C_{2}H_{6}}{C_{2}H_{2}\left(\mathrm{Feed}\right){-C}_{2}H_{2}}\right)\times 100$$

### 2.4. Catalyst Characterization

The catalyst was prepared using three different methods and examined using the following analyses. The surface morphology of the catalyst was analyzed using scanning electron microscopy (SEM, Keyence VE-9800, Osaka, Japan). Energy-dispersive X-ray spectroscopy (EDS, Genesis XM2, EDAX, NJ, USA) combined with SEM was performed to determine the distribution of Pd metal on the alumina support. To investigate the crystallite phase of the catalyst, X-ray power diffraction (XRD) was performed using Cu Ka radiation (40 kV, 15 mA, Rigaku Mini Flex 600, Tokyo, Japan). The diffraction patterns were recorded for 2θ values between 20° and 80° in 0.010° steps. Field-emission transmission electron microscope (FE-TEM) measurements were carried out using JEOL JEM-2010F (Tokyo, Japan) The crystal planes of Pd on the catalyst were confirmed using FE-TEM. Pd metal dispersion and crystallite size were analyzed using H_2_ chemisorption (Quantachrome ChemBET Pulsar, Boynton beach, FL, USA). H_2_ can undergo dissociative absorption on a Pd surface at room temperature. The dispersion of Pd on the surface of the catalyst was estimated from the amount of H_2_ adsorbed, assuming a Pd/H_2_ stoichiometry of 2. H_2_ chemisorption was conducted under the following conditions. The Pd/Al_2_O_3_ catalyst was reduced at a temperature of 573 K for 1 h, and hydrogen was flushed out under a N_2_ atmosphere for 30 min at the same temperature as a pretreatment [21]. The measurements were then performed at 300 K under a N_2_ atmosphere with a pure H_2_ pulse flow.

## 3. Results and Discussion

### 3.1. SEM

**Figure 2:**
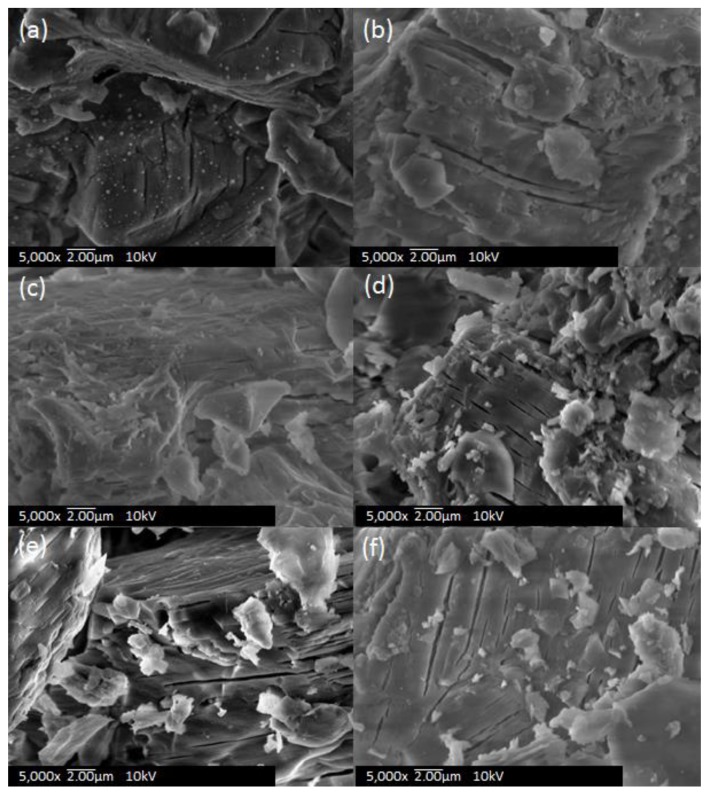
SEM images for Pd/Al_2_O_3_ catalysts: (**a**) fixed particle bed with plasma irradiation (PF)-1, (**b**) PF-2, (**c**), PF-3, (**d**) plasma spouted bed reactor (PS), (**e**) conventional method (CM)-500, and (**f**) CM-900.

Figure 2 shows SEM images of the catalysts prepared under all conditions. The surfaces were cracked at several places in all of the samples. However, PF-3 was relatively smooth, and few cracks existed. No significant differences were observed on the surface for CM-500 and CM-900, suggesting that higher temperatures had little effect on the surface morphology of the catalyst. Meanwhile, an obvious difference was observed in PF-1 when compared with other samples. Sphere-shaped objects on the surface that corresponded to Pd nanoparticles were formed. This phenomenon might have resulted from extremely high temperatures by the plasma due to the short distance between the waveguide and the particle bed. It was confirmed by EDS that Pd was uniformly distributed on the surface.

### 3.2. XRD

**Figure 3:**
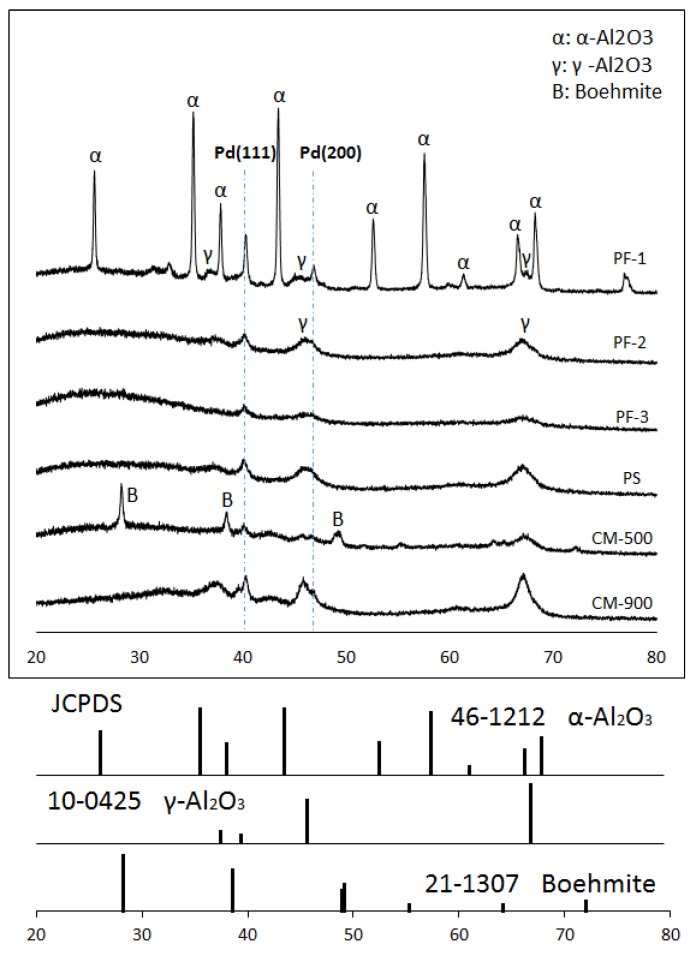
XRD patterns for Pd/Al_2_O_3_ catalysts.

Figure 3 shows the XRD spectra of the catalysts. The diffraction peaks (2θ) of XRD at 33.9°, 42.0°, 54.8°, and 60.8° corresponding to PdO (JCPDS card, file No. 47-1107) were not detected for all the plasma-treated samples, indicating that the plasma treatment successfully reduced PdO to Pd in a short period of time. The diffraction peaks at 40.1° and 46.7° corresponded to the Pd (111) and Pd (200) planes of the face-centered cubic (FCC) metal crystal of Pd (JCPDS card, file No. 46-1043), respectively.

PF-1 indicated a diffraction peak at 46.7°, while the other samples did not. For all of the catalysts, the crystalline phase of γ-alumina was detected at two values, specifically 47.4° and 67.2° (JCPDS card, file No. 10-0425). The diffraction peaks (2θ) at 25.6°, 35.1°, 37.8°, 43.4°, and 52.6° corresponded to the α-alumina peak (JCPDS card, file No. 46-1212). Only the PF-1 sample demonstrated an α-alumina phase. It is generally accepted that aluminum hydroxide cycles through various intermediate phases of alumina before it reaches its final state (α-alumina) with increasing temperature. Aluminum hydroxide also has numerous decomposition pathways after it has transitioned into α-alumina. These depend on several parameters including temperature, heating rate, and particle size [22,23]. The following pathway is most common for aluminum hydroxide decomposition and was also observed in our work when using the electric furnace:

Gibbsite(Al(OH)_3_)→boehmite(AlOOH)→γ-Al_2_O_3_→δ-Al_2_O_3_→θ-Al_2_O_3_→α-Al_2_O_3_.

Boehmite is formed by gibbsite at temperatures ranging between 100 °C and 300 °C. The decomposition of boehmite follows a series of transformations from γ-alumina to α-alumina with increasing temperature. The most thermodynamically stable phase of α-alumina is formed at temperatures greater than 1050 °C [24].

The peaks of γ-alumina coexisted with those of α-alumina in the PF-1 sample. However, the peaks corresponding to θ-alumina were not detected. Significantly, θ-alumina is an essential intermediate in the transition pathway from γ-alumina to α-alumina. A typical thermal treatment could not derive the direct phase transformation from γ-alumina to α-alumina without θ-alumina [25]. Meanwhile, these decomposition behaviors (γ→α) could be obtained by the use of a planetary ball mill [25,26,27,28]. Despite this, the rationale for the transition behavior of alumina remains to be proven. It was surmised that the plasma treatment and the conventional heating process using the electric furnace would have different pathways of pyrolysis for aluminum hydroxide.

### 3.3. TEM

**Figure 4:**
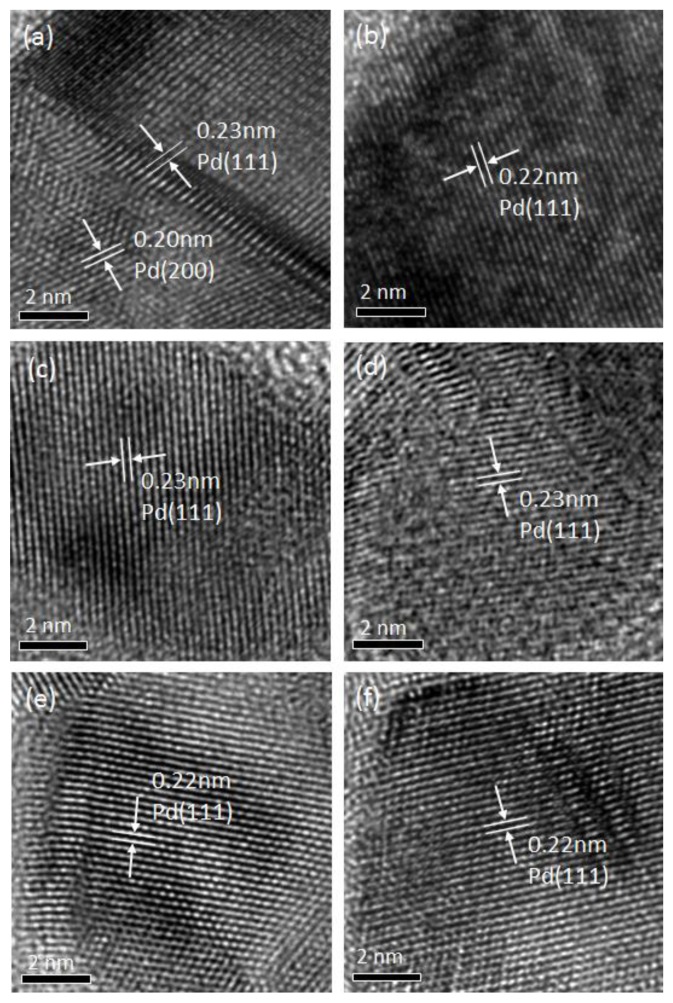
TEM images for Pd/Al_2_O_3_ catalysts: (**a**) PF-1, (**b**) PF-2, (**c**) PF-3, (**d**) PS, (**e**) CM-500, and (**f**) CM-900.

High-resolution TEM images of the catalysts are shown in Figure 4. From the images, the lattice plane spacings of 0.20 nm and 0.22–0.23 nm corresponded to the (200) plane and the (111) plane of Pd, respectively. All samples except PF-1 showed only the Pd (111) plane. Considered together with the XRD results above, the Pd (200) plane and the Pd (111) plane were obtained for PF-1. It is generally accepted that peaks corresponding to the (100) plane could be offset by that of the (200) plane due to destructive interference with the FCC structure. A square symmetry for the spots represents Pd (100) facets [12]. In this respect, the Pd (200) plane was regarded as the Pd (100) plane observed in PF-1. That is, PF-1 had both Pd (100) and Pd (111) simultaneously. It was reported that precursors such as Na_2_PdCl_4_ are typically required to obtain selective formations of Pd (111) and Pd (100) [11,29]. Thus, plasma treatment selectively forms Pd crystals independently of its precursors.

### 3.4. Acetylene Conversion

**Figure 5:**
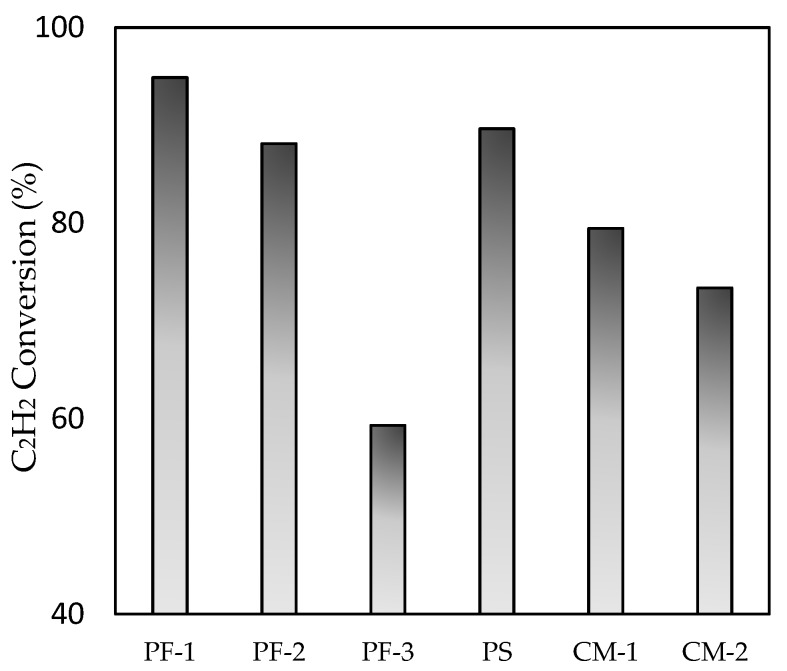
C_2_H_2_ conversion for Pd/Al_2_O_3_ catalysts.

C_2_H_2_ conversion of the catalysts prepared under all conditions is shown in Figure 5. Table 2 indicates the Pd metal dispersion, the crystallite size of Pd, and the metal surface area. From the H_2_ chemisorption results, PF-1 showed the greatest Pd dispersion, and PF-3 showed the lowest. Generally, high metal dispersion on the catalyst equates to high-performance reactions. The results of the C_2_H_2_ conversion are consistent with the results of H_2_ chemisorption. For the thermal treatment, high temperature caused metal sintering, leading to low dispersion [30]. Therefore, the lower C_2_H_2_ conversion of CM-900 compared with that of CM-500 might be derived from the sintering under high-temperature conditions. Considering the results obtained here, the catalyst prepared using the plasma fixed bed opposed typical results. The conversion of C_2_H_2_ increased as the distance of the waveguide and particle bed decreased (i.e., the order of C_2_H_2_ conversion was PF-1 > PF-2 > PF-3). As shown in the XRD data, the detection of α-alumina within the PF-1 sample indicated that the treated temperature was greater than 1050 °C. A shorter distance could provide more marked effects of the plasma and the gas temperature on the preparation. In general, C_2_H_2_ conversion decreases as temperature increases as a result of sintering. However, even at extremely high plasma temperatures, the plasma treatment had a positive effect on Pd dispersion. Plasma effectiveness on the metal dispersion of the catalyst has also been reported in other research [31,32,33]. The distance from the plasma irradiation (PF-3) had a marginal effect on the catalyst. Therefore, it is reasonable to indicate that the plasma treatment enhanced the distribution of Pd nanoparticles on the support by preventing the particles from migrating, leading to sintering.

### 3.5. Ethylene and Ethane Selectivity

**Figure 6:**
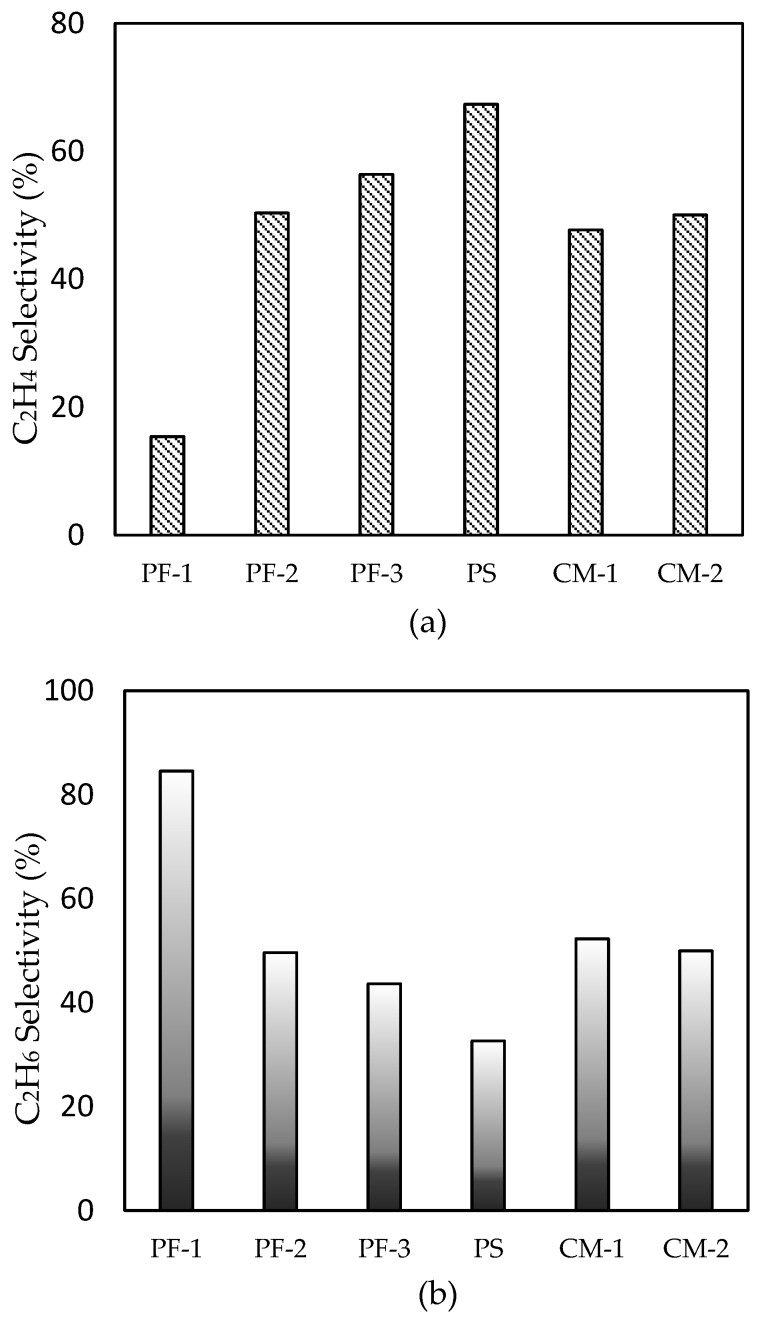
(**a**) C_2_H_4_ selectivity and (**b**) C_2_H_6_ selectivity for Pd/Al_2_O_3_ catalysts.

C_2_H_4_ and C_2_H_6_ selectivity for all samples are shown in Figure 6a,b, respectively. In Figure 6a, the catalysts prepared using the fixed bed with plasma irradiation (PF-2, PF-3, PS) showed greater catalytic performance in the C_2_H_4_ selectivity than those prepared using conventional thermal treatment. The plasma spouted bed-treated catalyst showed the highest ethylene selectivity. In Figure 6b, the difference in C_2_H_6_ selectivity between PF-1 and all other samples was significant. The longer the distance between the waveguide and the particles, the lower the C_2_H_6_ selectivity (PF-3 < PF-2 < PF-1). For PF-1, a remarkably high hydrogenation of C_2_H_4_ was observed when compared with other samples. This catalytic performance might be derived from the formation of the Pd (100) plane. This has been widely studied for various catalytic reactions (e.g., alkene hydrogenation, formic acid oxidation, and oxygen reduction reactions) for Pd (111) and Pd (100) [10,11,12]. In particular, Pd (100) has an effect on the consecutive hydrogenation of acetylene to ethane as compared with that of the Pd (111) plane [8,9]. Since the surface energy of Pd (100) is greater than that of Pd (111) [29,34], ethylene could be strongly attached to the Pd metal, promoting further hydrogenation of ethylene to ethane. From the XRD spectra and the TEM images, the Pd (100) plane was clearly verified for PF-1. A greater selectivity of C_2_H_6_ could be elucidated by the formation of the Pd (100) plane. As a consequence, the plasma treatment had the potential to induce specific modification of the catalyst for certain catalytic reactions as compared with conventional preparation methods.

### 3.6. Comparison of the Fixed Bed with Plasma Irradiation and the Plasma Spouted Bed

As mentioned above, the catalyst prepared by the fixed bed with plasma irradiation showed lower catalytic performance in the C_2_H_4_ selectivity than that prepared by the plasma spouted bed. This is illustrated by the significant difference in the formation of Pd (100) that was observed in PF-1. The plane of Pd (100) was not detected for PS, even when in close proximity to the waveguide. Moreover, for the samples without the Pd (100) plane (PF-2 and PF-3), C_2_H_4_ selectivity was also inferior to those of the plasma spouted bed. In our previous work, high C_2_H_4_ selectivity for PS could be explained by a strong interaction between the Pd metal and the alumina support. Regarding the surface energy, the strong interaction could encourage the Pd metal to easily desorb ethylene [35]. When considering the treatment conditions of PS and PF, the most remarkable difference was in the temperature fluctuation of the particles. The fixed bed with plasma irradiation could make the particle temperature constant. That is, the temperature was held constant from the beginning to the end during treatment. On the other hand, the particle temperature fluctuated due to the circulation in the bed. It remains unclear how the particles are affected by these processes. However, we may conclude that these effects are involved in the modification of phase components and induce the strong metal–support interaction (SMSI). In conclusion, an intensive effect of the plasma in the proximity of the irradiation could develop the formation of different crystal phases, and the spouted bed could control those effects by modulating temperature fluctuations.

## 4. Conclusions

A fixed bed with microwave-induced plasma jet irradiation was applied for a Pd/Al_2_O_3_ catalyst’s preparation. A high dispersion of Pd metal on the support was successfully fabricated in a short period of time. From the XRD spectra and the TEM images, the Pd (100) plane coexisted with the Pd (111) plane, and a direct decomposition pathway of alumina (γ→α) without the essential intermediate, θ-alumina, was observed in PF-1. Concerning the catalytic performance of the acetylene hydrogenation, the plasma-treated catalysts (PF-1, PF-2, and PS) with a shorter distance between the waveguide and the particles exhibited greater activity when compared with the furnace-treated catalysts. This was due to the smaller size of the Pd metal because the plasma treatment had a positive effect on preventing Pd nanoparticles from sintering. However, the highest selectivity of ethane for PF-1 could be explained by the creation of a higher surface energy in the Pd (100) plane obtained only for this condition. The effectiveness of plasma irradiation on the growth of the different crystal face of Pd and phase transition of alumina, as well as preparing the catalyst, was put into perspective by comparing it to conventional thermal treatment.

## Figures and Tables

**Table 1:** Treatment conditions of the fixed bed with plasma irradiation and the plasma spouted bed for catalyst preparation.

Catalyst	Power (W)	Distance ^1^ (cm)	Ar Flow Rate (L/min)	H_2_ Flow Rate (mL/min)	Treatment Time (min)	Mass (g)
PF-1	270	3.0	2.5	60	7	1.0
PF-2	270	5.0	2.5	60	15	1.0
PF-3	270	7.0	2.5	60	15	1.0
PS	270	1.0	2.5	60	15	1.0

^1^ The distance from the waveguide and the particle bed shown in Figure 1.

**Table 2:** H_2_ chemisorption results.

Catalyst	Avg. Crystallite Size of Pd (Å)	Metal Surface Area (m²/g Sample)	Dispersion ^1^ (%)
PF-1	14.8	1.13	25.3
PF-2	16.2	1.02	22.9
PF-3	25.5	0.65	14.8
PS	16.2	1.03	23.2
CM-500	19.2	0.86	19.6
CM-900	22.1	0.75	17.0

^1^ The fraction of Pd atoms exposed to the surface of the catalyst.
